# Improving Single-Cell Encapsulation Efficiency and Reliability through Neutral Buoyancy of Suspension

**DOI:** 10.3390/mi11010094

**Published:** 2020-01-15

**Authors:** Hangrui Liu, Ming Li, Yan Wang, Jim Piper, Lianmei Jiang

**Affiliations:** 1 ARC Centre of Excellence for Nanoscale BioPhotonics, Department of Physics and Astronomy, Macquarie University, Sydney, NSW 2109, Australia; hangrui.liu@hdr.mq.edu.au (H.L.); yan.wang24@students.mq.edu.au (Y.W.); 2 School of Engineering, Macquarie University, Sydney, NSW 2122, Australia; 3 ARC Centre of Excellence for Nanoscale BioPhotonics, Department of Molecular Sciences, Macquarie University, Sydney, NSW 2109, Australia

**Keywords:** single-cell analysis, droplet microfluidics, encapsulation efficiency, OptiPrep™, neutral buoyancy

## Abstract

Single-cell analysis is of critical importance in revealing cell-to-cell heterogeneity by characterizing individual cells and identifying minority sub-populations of interest. Droplet-based microfluidics has been widely used in the past decade to achieve high-throughput single-cell analysis. However, to maximize the proportion of single-cell emulsification is challenging due to cell sedimentation and aggregation. The purpose of this study was to investigate the influence of single-cell encapsulation and incubation through the use of neutral buoyancy. As a proof of concept, OptiPrep™ was used to create neutrally buoyant cell suspensions of THP-1, a human monocytic leukemia cell line, for single-cell encapsulation and incubation. We found that using a neutrally buoyant suspension greatly increased the efficiency of single-cell encapsulation in microdroplets and eliminated unnecessary cell loss. Moreover, the presence of OptiPrep™ was shown to not affect cellular viability. This method significantly improved the effectiveness of single-cell study in a non-toxic environment and is expected to broadly facilitate single-cell analysis.

## 1. Introduction

Single-cell analysis has been attracting great interest from both academia and industry as a powerful technique in the field of medical diagnosis, tissue engineering, and cell biology. Conventional biological assays focus on the signals based on large cell populations that normally provide average information about hundreds of thousands of cells; thus the existence of cell-to-cell variations and the intrinsic and inherent properties of cell populations are obscured [1,2]. Unlike bulk population analysis, single-cell analysis allows in-depth profiling of cell populations at single-cell resolution and uncovering of rare cell subpopulations. Furthermore, individual cells of interest can be sorted from the majority of the population for further studies. This technology has been demonstrated as a powerful tool to investigate cancer biology, by profiling marked heterogeneity, development of new diagnostics, and personalized medicine.

Traditional flow cytometry is mostly used in single-cell analysis to scrutinize heterogeneous cell populations in a high throughput and multiplexing manner [3,4]. However, some limitations have restricted its wide application for single-cell analysis. Fluorescence-activated cell sorting (FACS) mainly relies on cell-surface markers for detection, which curb the assay of intracellular interactions, secretions of metabolites, and genetic materials. Moreover, flow cytometry allows only single time point measurements, therefore, the same single cells can neither be observed over long periods nor suitable for repetitive measurements [2,5].

As an alternative, microfluidic droplet-based assays are fast, cost-effective, and high-throughput methods becoming valuable tools for single-cell analysis. Compartmentalization of single cells in monodispersed droplets has various advantages for applications across biology and biomedicine, because: (1) microenvironments of cells can be uniformly created and maintained to harmonize the extrinsic influence on cells; (2) droplets provide a unique tool to link genotypes with phenotypes of cells through confinement, as cells and molecules secreted remain trapped inside the droplets and are analyzed and sorted in a timeframe [6]; (3) the secreted molecules from single compartmentalized cells quickly reach a detectable limit because of the small volume of the confinement, which enables the rapid detection of molecules of interest; and (4) encapsulated cells can be lysed by cell lysis buffer without membrane rupture, enabling biochemical and genetic analyses of intracellular contents of single cells based on released DNA or RNA amplified in the droplets.

When using microfluidic chips to encapsulate single cells into droplets, the majority of droplets either contain more than one cell or are empty. Although the frequency of droplet generation and their diameters are tunable to produce more microdroplets containing exactly one cell, cell encapsulation in droplets is a random process limited by the Poisson distribution that is hard to control. Statistical models are established to provide an understanding of the underlying processes and estimation of the relevant parameters, however, high-efficient, reliable, and repeatable control over the encapsulation of single cells in droplets is still difficult to achieve due to the instrumental errors and cell sedimentation and aggregation [7]. Cell sedimentation changes the uniformity of cell distribution within the suspension (in the syringe, tubing, and microchannels), and therefore, increases the chance of aggregation. Ultimately, sedimentation and the resulting aggregation decrease the reliability of the droplet generation system by causing syringe clogging and non-uniform cell count per droplet. Therefore, to achieve higher single-cell encapsulation efficiency and reliability, sedimentation effects must be suppressed in the microfluidic droplet systems.

A variety of methods have been developed to increase the efficiency of single-cell encapsulation in microdroplets. External forces, such as acoustic, magnetic, and optical forces, have been integrated with microfluidic platforms to localize single cells at the droplet formation region, which help to increase the single-cell encapsulation rate. However, cell growth conditions may deteriorate when exposed to external stress, and normally the throughput of external force-assisted droplet generation methods is lower by an order of magnitude than that of methods without the need of external forces (i.e., at kHz rates). Furthermore, external force-assisted methods necessitate more complex experimental setup. In addition to external forces, the intrinsic properties of fluidics in microchannels have been used to enhance single-cell encapsulation efficiency. For example, the introduction of inertial flow coupled with Dean flow can increase the fraction of droplets containing single cells to ~80% [7,8]. However, the deterministic methods merely suit the encapsulation of homogenous cells, and the velocity of cell perfusion must correspond to that of droplet formation, which requires a meticulous operation. Moreover, the flow rates are much higher than those typically used in microfluidics chips (i.e., 1 < Re < 300 and flow velocities on the order of ~0.1–1 m s^−1^) [7], and the increased fluid shear stress may induce cell death. 

Magnetic stirring has been often used to prevent cell sedimentation and aggregation [9,10]. However, this method may cause unstable cell volume fraction, cell damage, and cell physiological fluctuations. Compared to the magnetic stirring method, the use of biocompatible density-matching gradient reagents to balance the density of cell suspensions is simpler and gentler. There are a number of different ideal density gradient materials, like sucrose, salts, polysucrose, and colloidal silica [11]. The selection of optimum reagent usually depends on the applications. The optimum should not alter the conditions of cells or particles to be separated and should provide a reliable density range for specific types of suspending cells or particles. OptiPrep™ [12,13] is a non-ionic iodinated solution containing 60% (*w*/*v*) iodixanol with a molecular weight of 1550 in water. The density of OptiPrep™ is 1.320 ± 0.001 g/mL and the endotoxins are below 1.0 EU/mL. OptiPrep™ was first developed for clinical purposes in X-ray contrast imaging [12,14]. Due to its non-toxicity, low osmotic pressure, and no obvious penetration into large particles, OptiPrep™ is extensively used for the isolation of different types of molecules and cells, including peroxisomes, cytokine, DNA, viruses, organelles, exosomes, and a wide range of mammalian cells [13,15,16,17,18].

OptiPrep^TM^ shows advantages when comparing with other density-matching reagents, such as Nycodenz^®^ [19], Metrizamide [20], and Percoll^®^ [11,21]. For example, Nycodenz^®^ can cause possible contamination when isolating particles [21]; Metrizamide is less cell-friendly due to its ionic nature; and there are concerns about the toxic effects of Percoll^®^. In addition, the density of Percoll^®^ (1.13 g/mL ± 0.005 g/mL at 25 °C) is higher than that of OptiPrep™, which means that a larger amount of Percoll® is required to make solutions with the same density.

Therefore, its biocompatible and non-invasive properties make OptiPrep™ an ideal additive in the aqueous phase for droplet-based microfluidics. The use of OptiPrep™ has been demonstrated for increasing encapsulation efficiency of single THP-1 cells in microdroplets [22]. However, the concentration of OptiPrep™ used in the previous article [22] (i.e., 18%) has not been optimized. Moreover, it is noteworthy that time-dependent behaviors of OptiPrep™ and its effect on cell encapsulation efficiency [23] have been only investigated under limited conditions: relatively low cell concentration (3 × 10^6^ cells mL^−1^ ), certain cell types (adherent cells), specific period of time (trypsinization of cells), and limited environment (gel materials with higher viscosity). A detailed study about the impact of Optiprep^TM^ and cell density on the encapsulation ratio and cell viability in microdroplets has not yet been reported to date. 

In this work, we attempt to mitigate cell sedimentation and improve the efficiency of single-cell encapsulation in microdroplets through neutral buoyancy. Although researchers have mentioned the use of OptiPrep^TM^ for cell encapsulation by droplet-based microfluidics [24,25,26,27,28], to the best of our knowledge, this is the first detailed report quantitatively evaluating the effect of OptiPrep^TM^ on THP-1 cell encapsulation efficiency and cell viability in microdroplets.

## 2. Theory

### 2.1. Stokes’ Law 

In order to achieve the desired cell suspension dispersion and mitigate the effects of sedimentation, an appropriate medium density that is equivalent to the density of target cells is essential. Theoretical values for sedimentation velocities can be obtained using Stokes’ law [29,30,31], as:

(1) $$v=\frac{\left(\rho_{p}-\rho_{f}\right)gD_{p}^{2}}{18µ},$$

where v is the sedimentation velocity, g is the gravitational acceleration, *D_p_* is the particle diameter, $\rho_{p}$ and $\rho_{f}$ are the particle and fluid densities, respectively, and µ is the fluid viscosity.

From Equation (1), we know that the sedimentation velocity will increase with effective particle diameter and this indicates that aggregation of cells results in increased sedimentation velocity.

### 2.2. Poisson Distribution

Here, the Poisson distribution is used as an informative predictor for the rate of single-cell encapsulation when the target cells are smaller than the droplets volumetrically and are distributed homogeneously in an aqueous solution. The Poisson distribution, which is a discrete probability distribution, has been used to calculate the probability of a single cell in one droplet during encapsulation, assuming there is random dispersion of cells in the sample and constant flow velocity (shown in Table S1). The use of OptiPrep™ can achieve uniform suspension of cells in the sample by tuning the aqueous density to that of cells. The probability of one droplet containing *k* cells can be dictated by

(2) $$P\;\left(k,\;\lambda \right)=\frac{\begin{array}{c} \lambda^{k}e^{-\lambda } \end{array}}{k!},$$

where *λ* is the average number of cells per droplet,

(3) $$\lambda =C\cdot V_{D},$$

*C* is the concentration of cells in aqueous solution with unit of cells/mL, and $V_{D}$ is the volume of each droplet. By replacing λ in Equations (2) with (3), the probability of droplets containing *k* cells at different droplet sizes and cell concentrations can be calculated by Poisson distribution using MATLAB (MathWorks, Natick, MA, USA).

## 3. Materials and Methods

### 3.1. Device Design and Fabrication

The droplet-based microfluidic device used in this study consists of two inlets for the perfusion of disperse phase and continuous phase, connecting microchannels with an aspect ratio of height/width = 1:2 (height: ~40 µm; width: ~80 µm), a rectangular observation chamber of 2 × 0.65 cm, and one outlet (shown in Figure S1). The geometry we used here was T-junction, in which the oil flowed horizontally towards the observational chamber, and the aqueous phase flowed vertically and sheared into uniform droplets. This droplet-based microfluidic device was fabricated using standard soft-lithography techniques, including: (i) mask design via computer-aided design software; (ii) mylar mask printing; (iii) fabrication of the SU-8 (SU-8 2035 or 2050, MicroChem, Newton, MA, USA) master mold; (iv) casting of poly(dimethyl siloxane) (PDMS) (Sylgard 184, Dow Corning, Midland, MI, USA); and (v) air plasma treatment on the surfaces of the glass substrate and PDMS slabs for irreversible covalent bonding. 

### 3.2. Cell Culture and Preparation

The acute monocytic leukemia THP-1 cell line was obtained from CellBank Australia. Cells were cultured in a vertical T-75 flask filled with 12 mL of the complete growth medium: 90% RPMI-1640 (Sigma-Aldrich, St. Louis, MO, USA), 10% fetal bovine serum (FBS, Thermo Fisher Scientific, Waltham, MA, USA), and supplemented with 1% penicillin-streptomycin (Thermo Fisher Scientific, USA); and kept in an incubator (Thermo Fisher Scientific, USA) which provides sterile conditions at 37 ℃ with 5% carbon dioxide. THP-1 cells were inoculated in fresh complete growth medium at an initial concentration of 2 × 10^5^ cells/mL. The number and viability of THP-1 cells were measured by the Trypan blue-based TC-20 automated cell counter (Bio-Rad, Hercules, CA, USA). Normally, to acquire enough volume (e.g., 1 mL) of cell suspension (e.g., 6 × 10^6^), two flasks of cells are cultured for four days simultaneously, then spun down and suspended with fresh medium which adjusts the cell density to the desired value to be used before the viability drops down to 95%. When cell number and viability both satisfied the requirements, THP-1 cells were used to perform encapsulation in microfluidic droplets.

### 3.3. Encapsulation of Single Cells in Water-in-Oil Droplets

Oil phase, Novec™ 7500 Engineered fluid (3M, St. Paul, MN, USA) mixed with 2% Pico-Surf™ 1 (Sphere Fluidics, Cambridge, UK) as surfactant, and aqueous phase cells in culture medium and OptiPrep™ (Sigma-Aldrich, USA), were delivered via two syringe pumps (PHD 2000, Harvard Apparatus, Holliston, MA, USA; Chemyx, Fusion 200, Stafford, TX, USA) into the microchip to produce cell-encapsulated microdroplets. The fluorinated ethylene propylene (FEP) tubing (IDEX, Lake Forest, IL, USA), with an inner diameter of 0.5 mm, was used for connecting the syringes to the microchip inlets. The microfluidic chip was used to produce uniform cell-laden droplets of different sizes by tuning the flow rate of the oil phase and aqueous phase.

### 3.4. Measurement of Cell Density and Viability with the Presence of OptiPrep™

The density gradient centrifugation method is considered as a golden standard to measure individual cell weight [32]. Here, we drew on this idea and mixed different volumes of OptiPrep™ with culture medium to achieve four cell suspensions of different OptiPrep^TM^ concentrations: 4%, 8%, 12%, and 16%. Cells were spiked into the density-tuned media at the concentration of 3 × 10^6^ cells/mL and incubated for at least half an hour to record the cellular precipitation. 

Cells at the concentration of ~3 × 10^6^ cells/mL were used to create cell-laden droplets with the medium containing 0% OptiPrep™, 8% OptiPrep™, 13.2% OptiPrep™, and 16% OptiPrep™, individually. Cell viability was monitored after 12 h and 24 h culture at 37 °C with 5% carbon dioxide. For each time point, cells from three replicate experiments were measured. Then 50 μL of cell-laden droplets were pipetted and 2 μL Pico-Break™ (Sphere Fluidics, UK) were added and mixed well by inverting the centrifuge tube five times mildly. After that, the oil phase was spun down at 300× *g* for 1 min at room temperature and the supernatant containing cells was aspirated into a new centrifuge tube. Then, the viability assay kit for animal live and dead cells (Biotium, Hayward, CA, USA) containing 2 μM Calcein AM and 4 μM EthD-III was mixed with the released cells at room temperature for 20 minutes. After staining, the cells suspension was imaged by fluorescein isothiocyanate (FITC) channel to see the live cells (green) and mCherry channel to see the dead cells (red) by a fluorescence microscope, IX-83 (Olympus, Tokyo, Japan).

### 3.5. Measurement of OptiPrep™ Inducing Effect on Cell Encapsulation 

Cell suspensions without OptiPrep™ (0% OptiPrep™), with 8% OptiPrep™, 13.2% OptiPrep™, and 16% OptiPrep™ were spiked into the aqueous phase, respectively, and then cell encapsulation in microdroplets was performed immediately after the addition and mixing. At least one hundred measurements were performed in the observation chamber for each replicate, and a total of three replicates were conducted for each condition. The experimental results of cell encapsulation in microdroplets were analyzed by one-way analysis of variance (ANOVA) to determine whether a significant difference was present between with OptiPrep™ and without OptiPrep™. The experimental data were fitted to the theoretical values based on Poisson statistics to show the effect of OptiPrep™ on cell encapsulation. The ~81 µm diameter droplets were produced to encapsulate single cells at cell density of 2 × 10^6^, 4 × 10^6^, 6 × 10^6^, and 8 × 10^6^ cells/mL, respectively.

### 3.6. Imaging and Image Analysis

Videos of droplet formation were recorded with a high-speed camera (Phantom Miro M/R/ LC320S, Vision Research, Inc., Wayne, NJ, USA), and a digital camera (DS-Qi1Mc, Nikon, Tokyo, Japan) equipped on an inverted microscope (Eclipse Ti-U, Nikon, Tokyo, Japan) was used to capture images of cell-laden droplets. An observation chamber on the microfluidic chip (Figure S1C,D) was specifically designed for the imaging of droplets. The number of droplets was counted by programs written in MATLAB (Figure S2). In order to show the size distribution of droplets and cell encapsulation ratios at different cell and OptiPrep™ concentrations, random frames of at least 100 droplets in the chamber were chosen for counting and statistical analysis using ImageJ (National Institutes of Health (NIH), Bethesda, MD, USA) (see Figure S3). 

## 4. Results and Discussion

### 4.1. OptiPrep™ Effectively Mitigates Cell Sedimentation

**Figure 1:**
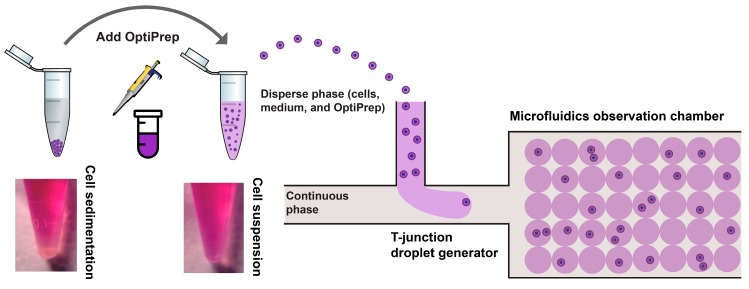
Schematic of a droplet-based microfluidic platform for highly efficient single-cell encapsulation using neutral buoyancy of suspension. Using an appropriate concentration of OptiPrep™ in culture medium prevents cell sedimentation.

In this work, we developed a medium density matching strategy to improve single-cell encapsulation efficiency and reliability through neutral buoyancy of cell suspension as shown in Figure 1. The two immiscible fluids (continuous phase: oil; dispersed phase: cell, medium, and OptiPrep^TM^) were simultaneously infused into the droplet generation part, a T-junction, of the fabricated microfluidic chip. The formation of droplets took place when shear force acting on the interface between two fluids overcame the interfacial force (shown in Video S1). Cells were encapsulated into microdroplets containing aqueous solutions surrounded by the oil phase.

Many cancer cells are known to aggregate and such aggregation phenomena were observed in many cancer-derived liquid biopsies [33,34]. Therefore, the suspension always consists of a large number of single cells as well as cell clusters. As seen in Figure S4A, we observed that cells were mostly clustered at the bottom of inlets and were not able to be perfused into microfluidic channels in the non-density matched suspension. This was due to the higher density of THP-1 cells (~1.05 g/mL) compared to the cell culture medium (~1.009 g/mL). Using the non-density matched suspension, the single-cell encapsulation ratio rarely matches the theoretical value of Poisson statistics. Cell suspension containing 0%, 4%, 8%, 12%, and 16% (*v*/*v*) OptiPrep™ were prepared individually to determine the appropriate medium density for preparing neutral buoyancy of THP-1 cell suspension. After 30 min, the THP-1 cells in whole culture medium (left tube) sedimented markedly, whereas density-matched THP-1 cells (right tube) were still well suspended, as shown in Figure 1. The density of culture media with 12% OptiPrep™ (1.046 g/mL) and 16% OptiPrep™ (1.059 g/mL) is closer to the density of THP-1 cells; neutral buoyancy of THP-1 cell suspension occurs in the media with OptiPrep™ between 12% and 16% (Figure S4B).

### 4.2. Efficienct Single Cell Encapsulation Using Neutral Buoyancy of Suspensions

Some researchers normally adopt the cell seeding of a low λ value to ensure that a large proportion of droplets contain only one cell, but it causes the majority of wasteful empty droplets. Unsurprisingly, the ratio of single-cell-encapsulated droplets increases at a higher λ value. For example, when λ = 1, the ratio of droplets containing a single cell is 36.8%, while the ratio of empty droplets and droplets containing more than one cell is 36.8% and 26.4%, respectively.

**Figure 2:**
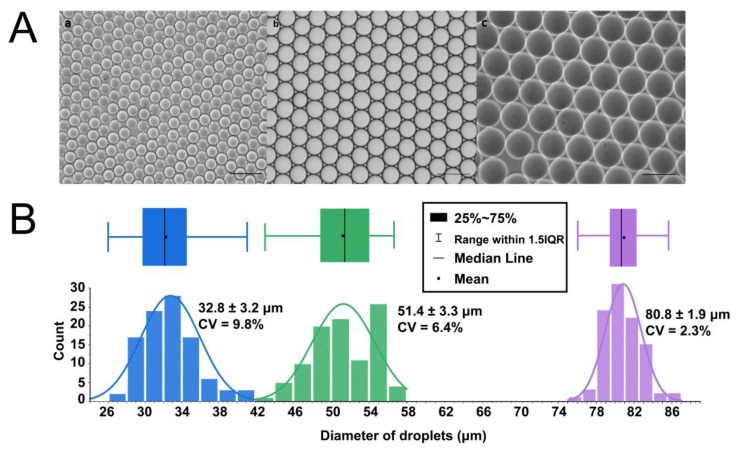
Microfluidic T-junction droplet-generator created uniform and stable droplets. (**A**) The morphology created droplets of three sizes: 32.8 μm, 51.4 μm, and 80.8 μm. Scale bar = 100 μm. (**B**) Plots of size distribution of droplets generated by the microfluidic chip. The limits of the box refer to the 25th and 75th percentiles, the cross line is the mean value, the black square is the median value, and the whiskers extend to 1.5 times the interquartile range (IQR). For each measurement, 100 droplets were counted.

Compared with the laborious change of cell density, droplet size is relatively easy to control. A microfluidic device with T-junction geometry was employed to encapsulate single THP-1 cells in monodisperse microdroplets (Figure 1). With constant flow rates, we produced highly monodisperse droplets, enabling highly reproducible reaction and analysis conditions. Fine tuning of the droplet size for a given channel geometry is accomplished by varying the fluid viscosity, aqueous flow rate or the overall flow rate. This also leads to variations in the drop production frequency. In Figure 2, it can be seen that we generated droplets with a mean diameter of 32.8 µm, and a standard deviation of 3.2%. By increasing the flow rate of the aqueous phase, we were able to generate droplets with a mean diameter of 51.4 µm, and a standard deviation of 6.4%. In order to maintain long-term cell culture in droplets, larger droplets with a mean diameter of 80.8 µm and a standard deviation of 1.9% were formed by decreasing the flow rate of the oil phase using the same device. Therefore, cells can be encapsulated with OptiPrep™ in highly monodisperse droplets for long-term cell culture and assays. 

**Figure 3:**
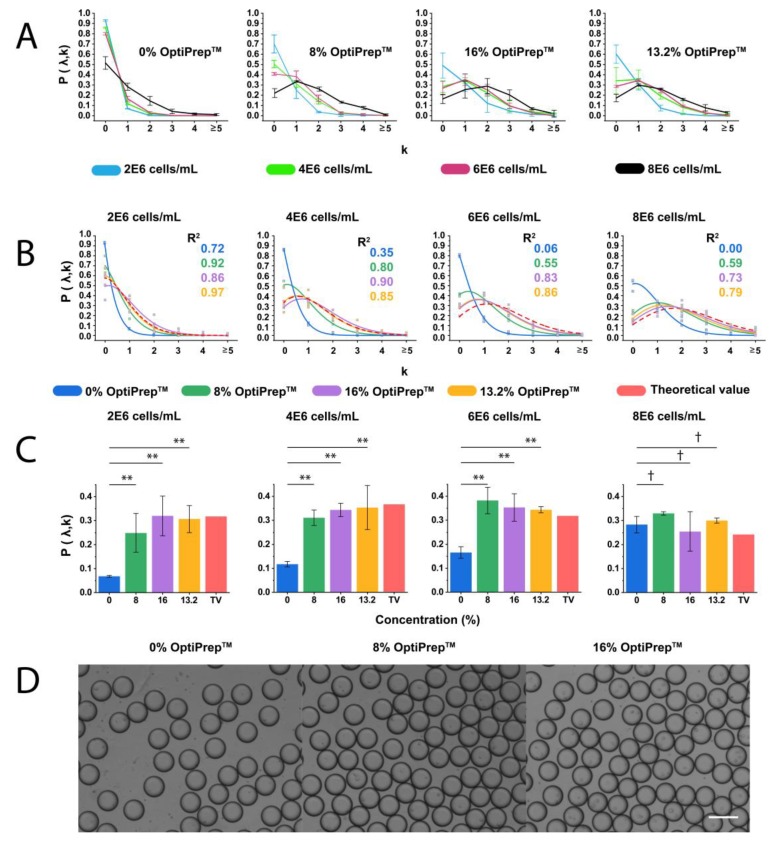
Cell encapsulation in microdroplets at different cell and OptiPrep^TM^ concentrations. (**A**) Plots of probability of THP-1 cell encapsulation in 81 µm droplets at different concentrations of OptiPrep™ (0%, 8%, 13.2%, and 16%) and cells (2 × 10^6^, 4 × 10^6^, 6 × 10^6^, and 8 × 10^6^ cells/mL). From the left to right: experimental values at 0% OptiPrep™, 8% OptiPrep™, 16% OptiPrep™, and optimized OptiPrep^TM^ (13.2%), and theoretical values predicted by Poisson distribution for cell encapsulation of 81 µm droplets. At least 100 droplets were counted for each measurement, and the experiment for each condition was duplicated three times. Error bars represent the standard deviation. The probability P (λ, k) indicates the proportion of droplets containing *k* cells (0, 1, 2, 3, 4, and ≧5). (**B**) The comparison between the theoretical and experimental results of cell encapsulation in microdroplets. Plots of the probability P (λ, k) values for droplets containing different number of cells (0, 1, 2, 3, 4, and ≧5) obtained from experiments and Poisson statistics are shown. R^2^ refers to the coefficient of determination of different groups fitting Poisson distribution. (**C**) ANOVA test results for the rate of single-cell encapsulation in droplets. A comparison between experimental values and theoretical value (TV) at different concentrations of OptiPrep^TM^ was performed for each cell concentration. ** represents *p* < 0.01; † means no significant difference. *n* = 3, error bars represent the standard deviation. (D) Photographs of cell-laden microdroplets generated with the presence of OptiPrep™ at different concentrations. From the top to bottom: 0% OptiPrep™, 8% OptiPrep™, and 16% OptiPrep™ in cell suspensions. Cell concentration was 4 × 10^6^ cells/mL. Scale bar = 100 µm.

The ratio of single-cell encapsulation within microdroplets of three different sizes (with a mean diameter of 32.8 μm, 51.4 μm, and 80.8 μm) at four different cell concentrations (2 × 10^6^ cells/mL, 4 × 10^6^ cells/mL, 6 × 10^6^ cells/mL, and 8 × 10^6^ cells/mL) was calculated by Poisson distribution (Figure S5 and Table S1). We used ~81 μm microdroplets to investigate the effect of OptiPrep™ concentration on cell-encapsulation efficiency, because it can achieve a higher ratio of droplets containing single cells and lower ratio of empty droplets compared to ~33 μm and ~51 μm droplets (see Figure S5). Moreover, a relatively lower concentration of cells is needed for ~81 μm microdroplets to study single-cell encapsulation. In theory, we assumed that cells are dispersed in culture medium uniformly and the maximum efficiency for encapsulating single cells is 36.6% in the ~81 µm droplets at the cell concentration of 4 × 10^6^ cells/mL. In practice, we tested the influence of density matching on the evenness of cell encapsulation rate by using cell suspension with different concentrations of OptiPrep^TM^ (Figure 3A). The single-cell encapsulation is below 10% in the non-density matched suspension at the cell concentration of 4 × 10^6^ cells/mL. The single-cell encapsulation rate increases to ~30% when cell suspension is prepared at the concentration of 8 × 10^6^ cells/mL. This is due to the significant variation between the calculated cell concentration and the real concentration after sedimentation. However, to achieve a high rate of single-cell encapsulation without density matching, an extremely high concentration of cells has to be used initially, which increases the risk of clogging in syringes and microchannels.

Figure 3A also displays the experimental results of cell encapsulation rate in ~81 µm droplets using non-sufficient density matching reagent (8% OptiPrep™). It shows that the single-cell encapsulation ratio is ~31.1 ± 3.2%, which is 5.6% lower than the predicted ratio by Poisson distribution. Although the increase of cell concentration from 4 × 10^6^ cells/mL to 6 × 10^6^ cells/mL can increase the single-cell encapsulation ratio, it leads to an increase ratio of droplets containing multiple cells. More specifically, the ratio of droplets containing single cells increases from 34.3 ± 2.8% to 35.3% ± 5.7%, while the ratio of droplets containing two cells increases from 22.2 ± 3.2% to 25.1 ± 5.7%. Therefore, cell concentration at 6 × 10^6^ cells/mL is not preferred for single THP-1 cell analysis in 81 µm droplets. In addition, a sudden increase in the ratio of droplets containing multiple cells was observed when cell concentration increased to 8 × 10^6^ cells/mL. These results could be attributed to a larger number of cells existing at the T-junction due to sedimentation, or cell clusters injected into the microchannel. 

In theory, the ratio of droplets containing cells obtained based on the neutral buoyancy of cell suspensions fits better than the ones predicted by Poisson distribution, and reduces the amount of OptiPrep™ to lessen its impact on cell physiology. Here we calculated and experimentally verified that the most appropriate concentration of OptiPrep™ to form THP-1 cell suspension is 13.2%. By using this concentration of OptiPrep™ and the cell density of 4 × 10^6^ cells/mL, experimentally obtained ratios of empty droplets (34.1 ± 12.3%), droplets with one cell (35.3 ± 9.2%), droplets with two cells (19.1 ± 2.6%), three cells (8.1 ± 6.6%), and four cells (2.4 ± 2.3%) are in good agreement with the theoretical ratios.

Figure 3B shows that the addition of 13.2% OptiPrep™ is optimal to predict the ratio of droplets containing different numbers of cells, as the experimental curves of the cell-laden droplet ratio under different conditions fit well to the theoretical values.

As shown in Figure 3C, in terms of single-cell droplet ratio, all groups but the 8 × 10^6^ cells/mL show a significant difference for 0% OptiPrep^TM^ vs. 8% OptiPrep^TM^, 0% OptiPrep^TM^ vs. 16% OptiPrep^TM^, and 0% OptiPrep^TM^ vs. 13.2% OptiPrep^TM^.

In this study, neutral buoyancy suspensions of THP-1 cells were prepared as a proof-of-concept. Other neutral buoyancy suspensions with specific entities, such as various cancer cells, bacteria, fungi, and particles can be calculated and prepared depending on the density of targets. 

### 4.3. Cell Viability and Proliferation with the Presence of OptiPrep™

**Figure 4:**
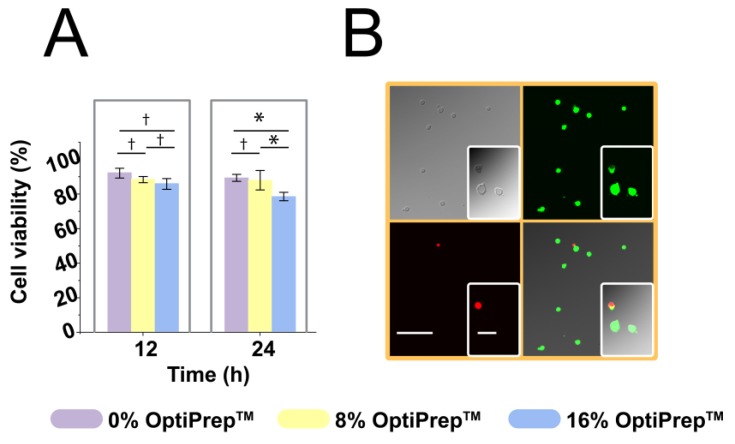
THP-1 cells maintained high viability with the presence of OptiPrep™ over 24 h inside the droplets. (**A**) Cell viability at two time points: 12 h and 24 h; * represents *p* < 0.05; † represents no significant difference; error bars represent the standard deviation. (**B**) Images showing cell viability. Measurement of cell viability was performed with the live/dead assay, live cells were stained with Calcein-AM (green) and dead cells with EtD-III (red); scale bar = 100 μm for images obtained by a 20× objective; scale bar = 10 μm for insets obtained by a 100× objective.

We examined whether a prolonged period of suspension and incubation would affect cellular viability. This was to ensure that suspending cells in a neutrally buoyant suspension is a feasible and stable approach for long-time single-cell assays. To test the viability of cells in the droplets after 12 h and 24 h culture, we removed the oil layer before performing the live/dead viability stains. As shown in Figure 4, 91.9 ± 3.0% cells remained alive after 12 h incubation and the cell viability decreased to 88.9% ± 2.0% after 24 h for the 0% OptiPrep™; while 88.1 ± 1.8% cells remained alive after 12 h incubation and slightly decreased to 87.5% ± 5.6% after 24 h with the presence of the 8% OptiPrep™. When the concentration of OptiPrep™ increased to 16%, the cell viability was only 85.6 ± 3.1% after 12 h incubation and significantly decreased to 78.1% ± 2.4% after 24 h. Statistical analysis shows that there is no significant difference in cell viability between different concentrations of OptiPrep™ after 12 h incubation (see Figure 4). However, after 24 h culture, both measurements of cell viability experienced a significant decrease when 16% OptiPrep™ was present.

Some factors might contribute to the more significant decrease in viability of cells cultured inside droplets: the main ingredient of Pico-Break™, PFOH (1H,1H,2H,2H-Perfluoro-1-octanol), imposed chemical stress on the cells; and the nutrient deficiency due to the dilution from OptiPrep™. However, these factors can be carefully controlled with finer operation during oil removing and regulating the droplets’ size in the droplet-based cell assay.

OptiPrep™ is a non-ionic solution of 60% iodixanol (*w*/*v*) in water. Materials such as sucrose and dextran have also been used to create density gradients for cell separations. In comparison to sucrose solutions at similar concentrations, solutions of OptiPrep™ had a much lower osmolarity and viscosity. The low osmolality of OptiPrep™ solutions allows for its use in cell separation. Dextran, a linear polymer of glucose, has also been used to increase solution density; however, it has a much higher viscosity at similar OptiPrep™ solution densities. Thus, OptiPrep™ has the advantages of both low osmotic pressure and low intrinsic viscosity. Preparing neutral buoyancy of suspensions with appropriate concentrations of OptiPrep™ opens up the possibility to improve single-cell encapsulation significantly without affecting cell viability.

## 5. Conclusions

We hypothesized that the influence of physical forces on cells within suspension would lead to stratification of concentrations found within the fluid, and that this would result in unreliable and inaccurate cell counts and manipulations. We reasoned that the single-cell encapsulation ratio and reliability over time could increase by mitigating these effects. We alleviated the negative influence of gravity by adding OptiPrep™ to achieve near neutral buoyancy within the suspension. Our hypothesis was confirmed by experimental results: density balancing of cell-based suspensions resulted in an increased ratio of single-cell encapsulation in microdroplets and enabled reliable encapsulation distribution that was close to the theoretical values.

We also investigated the effect of initial cell concentrations on single-cell encapsulation in microdroplets with OptiPrep™ of different concentrations. Furthermore, cell viability in microdroplets with the presence of various concentrations of Optiprep^TM^ has been investigated for the first time. This is a pioneering work on evaluating the influence of OptiPrep™ as a density-matching reagent on the efficiency of single-cell encapsulation and culture in microdroplets. It proves that this method can enhance the ratio of droplets encapsulated with single cells and has limited adverse effects on THP-1 cell viability when low concentrations of Optiprep^TM^ are used. Interestingly, middle-range concentrations of OptiPrep™ can also be used to increase cell encapsulation efficiency. However, given that this just reduces the sedimentation velocity of cells, the non-sufficient density matching reagent is only recommended for relatively short time of cell encapsulation. 

In conclusion, we demonstrated the feasibility of improving single-cell encapsulation efficiency and reliability using OptiPrep™. We expect this density-matching approach could open the door to realize highly efficient single-cell analysis in a milder and more cost-effective manner. We also envision that this approach can be widely used for a wide variety of applications involving three-dimensional (3D) bioprinting.

## Supplementary Materials

The following are available online at https://www.mdpi.com/2072-666X/11/1/94/s1. Figure S1: Photographs of the microfluidics chips and experimental setup, Figure S2: A photograph of droplets recognized by programs written by MATLAB, Figure S3: Actual images of cells encapsulated within microdroplets under different conditions, Figure S4: A photograph of cell sedimentation at the inlet of a microchannel, Figure S5: Plots of Poisson distribution for droplets of three different sizes, Table S1: Poisson statistics for different cell concentrations and droplet sizes, Table S2: Data collected from captured images of cell-laden droplets. The data obtained from three duplicates are shown, Table S3: Data collected from viability test. The data obtained from three duplicates are shown. Video S1: Droplet formation at the T-junction of a microfluidic droplet generator.
