# Supplementary material for: Improving Single-Cell Encapsulation Efficiency and Reliability through Neutral Buoyancy of Suspension
Source: Micromachines (Basel). 2020 Jan 15;11(1):94. doi: 10.3390/mi11010094 (PMC7019761; doi:10.3390/mi11010094)
Supplement: Supplementary file 1 [file micromachines-11-00094-s001.zip › micromachines-687474-spplementary/micromachines-687474-supplementary.docx]

Supplementary Materials: Improving Single-Cell Encapsulation Efficiency and Reliability through Neutral Buoyancy of Suspension

Hangrui Liu ^1^, Ming Li^2,^*, Yan Wang ^1^, Jim Piper ^1,^* and Lianmei Jiang ^3,^*

^1^ ARC Centre of Excellence for Nanoscale BioPhotonics, Department of Physics and Astronomy, Macquarie University, Sydney, NSW 2109, Australia

^2^ School of Engineering, Macquarie University, Sydney, NSW 2122, Australia

^3^ ARC Centre of Excellence for Nanoscale BioPhotonics, Department of Molecular Sciences, Macquarie University, Sydney, NSW 2109, Australia

***** Correspondence: ming.li@mq.edu.au (M.L.); jim.piper@mq.edu.au (J.P.); lianmei.jiang@mq.edu.au (L.J.); Tel.: +61 2 9850 9532 (M.L.); +61 2 9850 6369 (J.P.); +61 2 9850 8115 (L.J.).


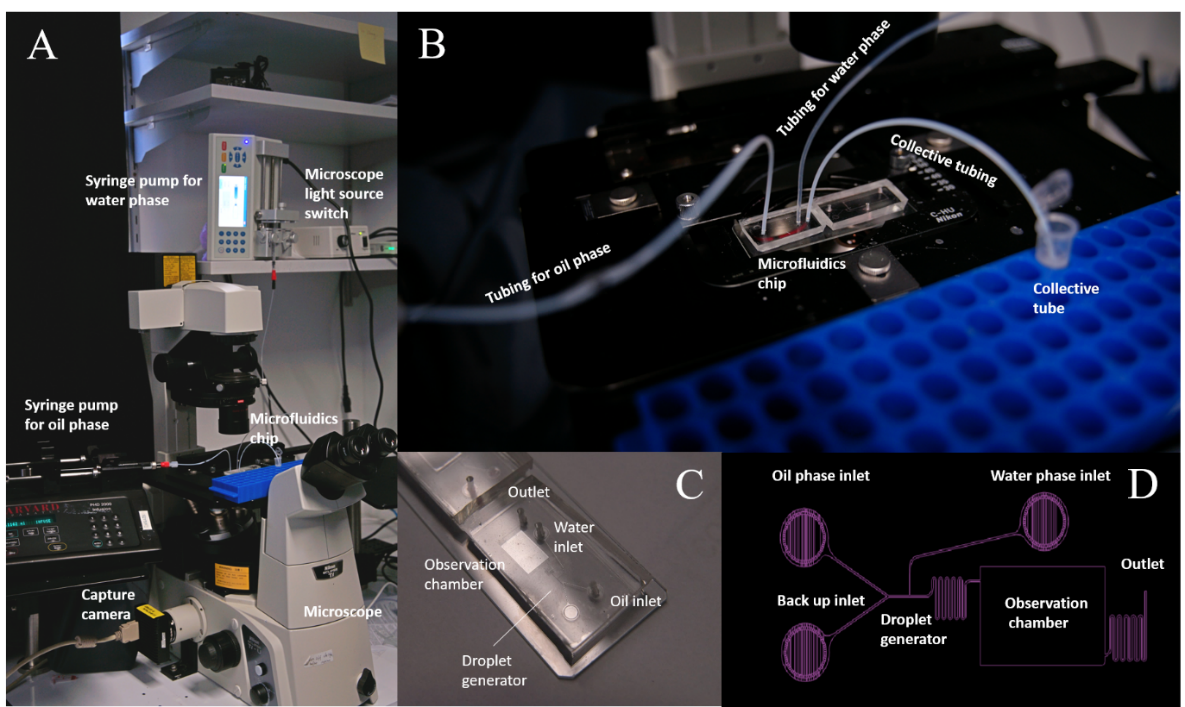


**Figure S1.** Photographs of the microfluidics chips and experimental setup. (**A**) A photograph of experimental setup. The external equipment required to generate cell-laden droplets include two syringe pumps for oil phase (continuous phase) and water phase (disperse phase), respectively, a microscope, a light source, a camera to capture images and videos, and a fabricated microfluidic chip. (**B**) An enlarged view of the device for droplet generation under the microscope. Three tubing for the injection of oil phase and water phase, and sample collection are shown. (**C**) The actual image of the microfluidic chip, which consists of two inlets for oil phase and a water phase, respectively, a T-junction to create microdroplets, an observation chamber, and an outlet. (**D**) The CAD file showing the design of the chip.


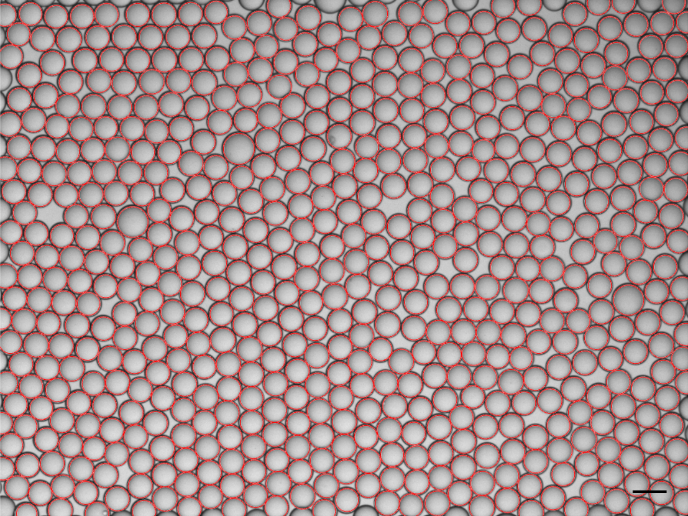


**Figure S2.** A photograph of droplets recognized by programs written by MATLAB. 81 µm droplets were captured and automatically calculated by programs written by MATLAB: count number = 582 droplets; scale bar = 100 µm.


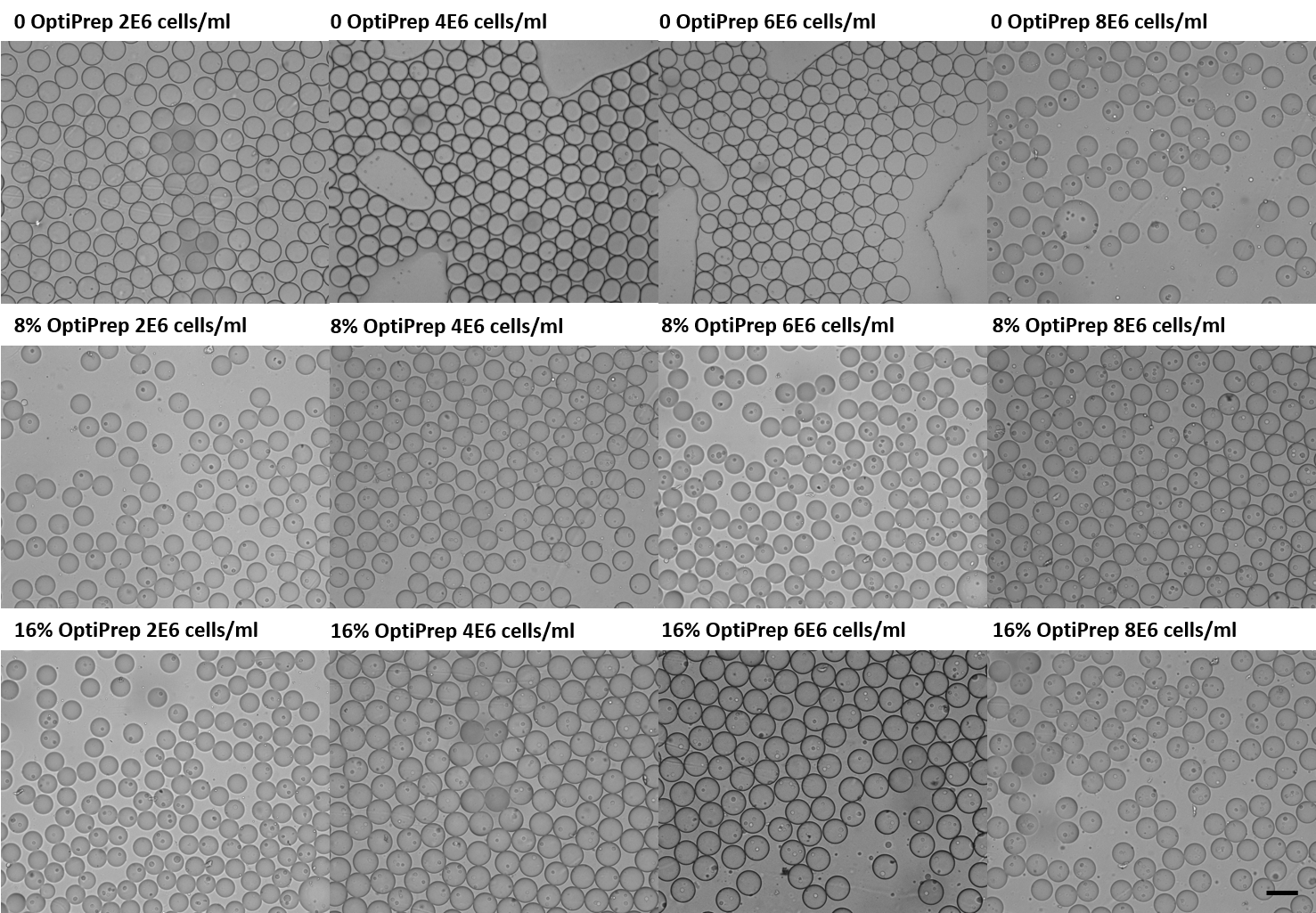


**Figure S3.** Actual images of cells encapsulated within microdroplets under different conditions. Experimental images of cell encapsulation in microdroplets under four different cell concentrations (2 × 10^6^, 4 × 10^6^, 6 × 10^6^ and 8 × 10^6^ cells/mL) and three different OptiPrep^TM^ concentrations (0%, 8% and 16%). Scale bar = 100 µm.


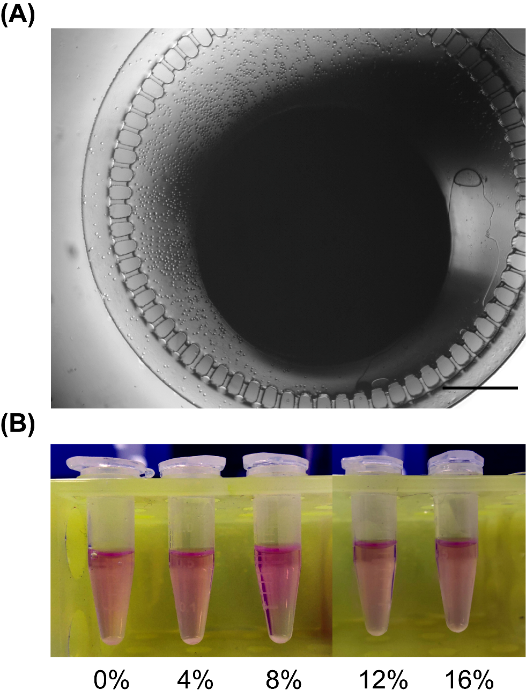


**Figure S4.** A photograph of cell sedimentation at the inlet of a microchannel. Cell sedimentation after 30 min in suspensions containing OptiPrep^TM^ at different concentrations. (**A**) A photograph of cell sedimentation at the inlet of a microfluidic channel. Scale bar = 500 µm. (**B**) Cell sedimentation in culture media with OptiPrep^TM^ at different concentrations of 0%, 4%, 8%, 12% and 16% (from left to right).


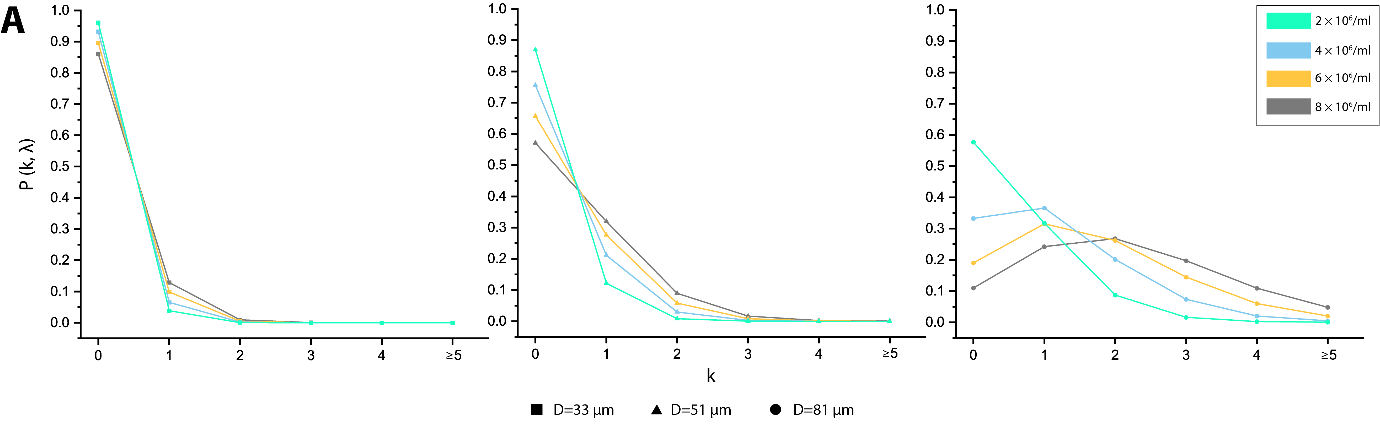


**Figure S5.** The plots of Poisson distribution for droplets of three different sizes (33, 51 and 81 μm). Four cell concentrations (2 × 10^6^, 4 × 10^6^, 6 × 10^6^ and 8 × 10^6^ cells/mL) were used to represent different λ values, and probabilities of droplets capturing different numbers of cells (0, 1, 2, 3, 4 and ≧ 5) were shown.

**Table S1.** Poisson statistics for different cell concentrations and droplet sizes.

| **Cell density (cell/mL)** | **Droplets Size (μm)** | **λ** | **Empty** | **1** | **2** | **3** | **4** | **5** |
| --- | --- | --- | --- | --- | --- | --- | --- | --- |
| 8.0E + 06 | 32.8 | 0.148 | 0.861 | 0.129 | 0.010 | 0.000 | 0.000 | 0.000 |
| 6.0E + 06 | 32.8 | 0.111 | 0.896 | 0.099 | 0.005 | 0.000 | 0.000 | 0.000 |
| 4.0E + 06 | 32.8 | 0.074 | 0.932 | 0.065 | 0.002 | 0.000 | 0.000 | 0.000 |
| 2.0E + 06 | 32.8 | 0.037 | 0.961 | 0.038 | 0.001 | 0.000 | 0.000 | 0.000 |
| 8.0E + 06 | 51.2 | 0.562 | 0.571 | 0.320 | 0.090 | 0.017 | 0.002 | 0.000 |
| 6.0E + 06 | 51.2 | 0.422 | 0.657 | 0.276 | 0.058 | 0.008 | 0.001 | 0.000 |
| 4.0E + 06 | 51.2 | 0.281 | 0.756 | 0.212 | 0.030 | 0.003 | 0.000 | 0.000 |
| 2.0E + 06 | 51.2 | 0.141 | 0.869 | 0.122 | 0.009 | 0.000 | 0.000 | 0.000 |
| 8.0E + 06 | 80.8 | 2.210 | 0.110 | 0.242 | 0.268 | 0.197 | 0.109 | 0.048 |
| 6.0E + 06 | 80.8 | 1.657 | 0.190 | 0.316 | 0.262 | 0.145 | 0.060 | 0.020 |
| 4.0E + 06 | 80.8 | 1.105 | 0.333 | 0.366 | 0.201 | 0.074 | 0.020 | 0.004 |
| 2.0E + 06 | 80.8 | 0.552 | 0.577 | 0.317 | 0.087 | 0.016 | 0.002 | 0.000 |

**Table S2.** The data collected from images of cell-laden droplets.

| **Name** | **Total** | **Zero** | **One** | **Two** | **Three** | **Four** | **Five or above five** |
| --- | --- | --- | --- | --- | --- | --- | --- |
| 0 OptiPrep 2E6 cells/mL A | 180 | 166 | 13 | 1 | 0 | 0 | 0 |
| 0 OptiPrep 2E6 cells/mL B | 140 | 131 | 9 | 0 | 0 | 0 | 0 |
| 0 OptiPrep 2E6 cells/mL C | 147 | 137 | 10 | 0 | 0 | 0 | 0 |
| 0 OptiPrep 4E6 cells/mL A | 166 | 142 | 21 | 3 | 0 | 0 | 0 |
| 0 OptiPrep 4E6 cells/mL B | 223 | 191 | 27 | 3 |  | 0 |  |
| 0 OptiPrep 4E6 cells/mL C | 259 | 223 | 27 | 8 | 1 | 0 | 0 |
| 0 OptiPrep 6E6 cells/mL A | 123 | 98 | 21 | 4 | 0 | 0 | 0 |
| 0 OptiPrep 6E6 cells/mL B | 187 | 148 | 35 | 3 | 1 | 0 | 0 |
| 0 OptiPrep 6E6 cells/mL C | 258 | 210 | 36 | 10 | 1 | 1 | 0 |
| 0 OptiPrep 8E6 cells/mL A | 163 | 82 | 41 | 24 | 9 | 4 | 3 |
| 0 OptiPrep 8E6 cells/mL B | 119 | 66 | 38 | 12 | 2 | 1 | 0 |
| 0 OptiPrep 8E6 cells/mL C | 187 | 83 | 52 | 35 | 10 | 4 | 3 |
| 8% OptiPrep 2E6 cells/mL A | 205 | 138 | 52 | 6 | 5 | 2 | 2 |
| 8% OptiPrep 2E6 cells/mL B | 116 | 73 | 38 | 5 | 0 | 0 | 0 |
| 8% OptiPrep 2E6 cells/mL C | 114 | 91 | 19 | 4 | 0 | 0 | 0 |
| 8% OptiPrep 4E6 cells/mL A | 258 | 141 | 73 | 29 | 6 | 5 | 4 |
| 8% OptiPrep 4E6 cells/mL B | 156 | 75 | 54 | 23 | 4 | 0 | 0 |
| 8% OptiPrep 4E6 cells/mL C | 152 | 74 | 46 | 24 | 6 | 2 | 0 |
| 8% OptiPrep 6E6 cells/mL A | 234 | 98 | 75 | 48 | 7 | 4 | 2 |
| 8% OptiPrep 6E6 cells/mL B | 150 | 59 | 60 | 24 | 5 | 2 | 0 |
| 8% OptiPrep 6E6 cells/mL C | 143 | 59 | 61 | 21 | 2 | 0 | 0 |
| 8% OptiPrep 8E6 cells/mL A | 293 | 94 | 121 | 45 | 7 | 2 | 24 |
| 8% OptiPrep 8E6 cells/mL B | 137 | 23 | 46 | 38 | 18 | 12 | 0 |
| 8% OptiPrep 8E6 cells/mL C | 139 | 35 | 46 | 34 | 16 | 8 | 0 |
| 16% OptiPrep 2E6 cells/mL A | 85 | 51 | 20 | 3 | 6 | 3 | 2 |
| 16% OptiPrep 2E6 cells/mL B | 95 | 34 | 38 | 20 | 3 | 0 | 0 |
| 16% OptiPrep 2E6 cells/mL C | 151 | 77 | 49 | 18 | 6 | 1 | 0 |
| 16% OptiPrep 4E6 cells/mL A | 145 | 50 | 54 | 28 | 8 | 2 | 3 |
| 16% OptiPrep 4E6 cells/mL B | 142 | 33 | 45 | 31 | 22 | 7 | 4 |
| 16% OptiPrep 4E6 cells/mL C | 129 | 36 | 44 | 33 | 9 | 6 | 1 |
| 16% OptiPrep 6E6 cells/mL A | 114 | 36 | 37 | 22 | 14 | 5 | 0 |
| 16% OptiPrep 6E6 cells/mL B | 124 | 24 | 52 | 38 | 9 | 1 | 0 |
| 16% OptiPrep 6E6 cells/mL C | 114 | 34 | 36 | 29 | 11 | 4 | 0 |
| 16% OptiPrep 8E6 cells/mL A | 203 | 30 | 39 | 62 | 44 | 16 | 12 |
| 16% OptiPrep 8E6 cells/mL B | 116 | 25 | 26 | 41 | 17 | 7 | 0 |
| 16% OptiPrep 8E6 cells/mL C | 115 | 15 | 40 | 24 | 28 | 8 | 0 |
